# Editorial: Advanced bioprocessing strategies for tissue engineering and biomimetic modelling applications

**DOI:** 10.3389/fbioe.2026.1826813

**Published:** 2026-03-16

**Authors:** João C. Silva, Carlos A. V. Rodrigues, Eirini Velliou, Diana Massai

**Affiliations:** 1 Department of Bioengineering, iBB-Institute for Bioengineering and Biosciences, Instituto Superior Técnico, Universidade de Lisboa, Lisboa, Portugal; 2 Associate Laboratory i4HB—Institute for Health and Bioeconomy, Instituto Superior Técnico, Universidade de Lisboa, Lisboa, Portugal; 3 Cell4Food, Matosinhos, Portugal; 4 Centre for 3D models of Health and Disease, Division of Surgery and Interventional Science, University College London, London, United Kingdom; 5 Department of Mechanical and Aerospace Engineering and PolitoBIOMed Lab, Politecnico di Torino, Turin, Italy; 6 Interuniversity Center for the Promotion of the 3Rs Principles in Teaching and Research, Turin, Italy

**Keywords:** biomaterial scaffolds, bioprocess engineering, bioreactors, in silico simulations, in vitro models, physical stimuli, regenerative medicine, stem cells

Tissue Engineering (TE) strategies have emerged as promising approaches for the regeneration/replacement of injured or diseased tissues and organs. Although significant progress has been achieved in the field, leading to the development of advanced tissue models, issues such as scalability, reproducibility, and regulatory compliance continue to hinder widespread clinical adoption. As a result, TE approaches have been mainly applied in the development of advanced biomimetic *in vitro* models for basic and preclinical research, typically in disease modeling, drug discovery, and toxicological screening applications. In this context, the application of additive fabrication technologies (e.g., 3D printing, bioprinting), is emerging as a powerful strategy to generate tissue constructs with enhanced architectural complexity and structural fidelity. Furthermore, advanced bioprocessing strategies, particularly bioreactor-based systems, are critical for the scalable and reproducible production of functional tissue-engineered constructs. Bioreactors provide controlled dynamic culture conditions that enable precise regulation of physico-chemical parameters, promoting enhanced mass transfer, high cell viability, and construct structural stability, while also enhancing extracellular matrix (ECM) deposition and tissue maturation (Sarkar et al., 2023). Notably, these systems are key for the development of physiologically relevant biomimetic models for investigating disease pathophysiology (e.g., tumor progression), as well as their response to treatments under controlled “*in vivo*-like” dynamic conditions. In parallel, increasingly sophisticated *in silico* frameworks, leveraging recent advances in computational modeling and artificial intelligence (AI), are essential for optimizing, controlling, and automating TE workflows. Importantly, advanced *in vitro* and *in silico* platforms have been increasingly acknowledged by regulatory agencies, including the FDA, as viable alternatives to animal testing.

Collectively, the above approaches are expected to accelerate the translational pipeline and play a critical role in enabling the safe and effective clinical implementation of regenerative medicine strategies. Therefore, this Research Topic focused on highlighting innovative contributions (including five original research articles and one review) covering the development of experimental bioreactor-based and *in silico* simulation strategies towards improved tissue regeneration settings and highly reliable disease models.

Bioreactor-based processes comprise the most efficient strategy to achieve the required cell numbers for clinical application. For example, despite equine cord blood mesenchymal stromal cells (eCB-MSC) have been found to be a promising treatment for equine joint repair upon traumatic injuries and osteoarthritis, challenges associated with process scalability remain. To address such limitations, Roberts et al. developed a scale-up strategy for eCB-MSCs consisting of a microcarrier-based culture using a 3L computer-controlled Vertical-Wheel® (VW) bioreactor (Roberts et al.). This work, which was the first known published study expanding any cell type in the 3L VW bioreactor, demonstrated that the developed scale-up bioprocess can achieve clinically relevant eCB-MSC numbers without any adverse effects on cell phenotype and function, showing high potential for veterinary regenerative medicine applications in equine health. As mentioned above, bioreactors can also be employed as controlled dynamic culture platforms for the preclinical validation of advanced therapy medicinal products (ATMPs). Accordingly, Gomes et al. reported the applicability of automated microbioreactors for the maintenance of highly viable human induced pluripotent stem cell (hiPSC)-derived neurospheroids, which, through the parallelization of the culture setup, could serve as a reliable and reproducible screening platform to address the transduction and tropism of recombinant adeno-associated viruses (Gomes et al.).

The integration of *in silico* simulations with 3D printed anatomical models has been recognized as a promising solution to enhance the precision of current surgical procedures. The study from Yang et al. is an excellent example of this successful combination, in which the authors used CT scan data from patients with complex spinal deformities to create personalized digital reconstructions and 3D printed spinal models and guides to simulate and optimize screw placement and osteotomy procedures (Yang et al.). Digital twin models are also being used to guide bioreactor design and optimize physical stimulation protocols, reducing experimental time and costs. Following this approach, Meneses et al. introduced *JANUS* - an open-source 3D printable perfusion bioreactor and a numerical model-driven design strategy for TE applications (Meneses et al.). The proposed prototype can deliver simultaneously capacitive-coupled electrical field and fluid-induced shear stress stimulations in an automated and controlled manner. Moreover, both stimulation systems were validated empirically in agreement with *in silico* predictive models, and the developed bioreactor was shown to support the viability of human osteoblasts cultured on 3D polycaprolactone scaffolds under electrical stimulation. In a different study, Fragomeni et al. developed a novel dynamic perfusion bioreactor designed to promote the effectiveness of ovarian tissue strip culture over static culture methods by exploiting the synergic tissue response to enhanced oxygen transport and adequate mechanical (fluid dynamic shear stress and direct compressive strains) stimulation (Fragomeni et al.). Remarkably, the perfusion bioreactor culture resulted in enhanced follicle viability, activation, and development through a closer mimicry of the ovarian physiological microenvironment. In addition to the types of physical stimuli mentioned above, pulsed electromagnetic field (PEMF) stimulation has been shown to promote tissue regeneration, particularly in the treatment of bone fractures and cartilage defects. Nevertheless, the effects of PEMF, particularly the underlying biological mechanisms related to the activation of specific signaling pathways, are not yet fully understood or correlated with specific PEMF stimulation protocols. Masante et al. addressed this topic by providing a comprehensive overview of the current state of the art of *in vitro* and *in vivo* experimental settings investigating the effects of PEMF stimulation on bone and cartilage regeneration, highlighting the great variability in the protocols employed across studies (Masante et al.). Importantly, the authors introduced a pioneer quantitative descriptor for PEMF stimulation (e.g., PEMF dose), enabling comparisons across different published protocols and helping in understanding the correlation between applied PEMF stimulation parameters and biological effects. Such knowledge gain will foster the refinement of future research and support the development of standardized guidelines for improved PEMF treatments.

In conclusion, we are proud to present our Research Topic to the readers of Frontiers in Bioengineering and Biotechnology. Collectively, the contributions in this Research Topic highlight the pivotal role of bioprocess engineering and *in silico* methodologies in driving the development of clinically translatable TE and biomimetic modeling platforms, paving the way for more predictive, scalable, and regulatory-aligned regenerative medicine strategies.
